# Combined effect of metformin with ascorbic acid versus acetyl salicylic acid on diabetes-related cardiovascular complication; a 12-month single blind multicenter randomized control trial

**DOI:** 10.1186/s12933-017-0584-9

**Published:** 2017-08-14

**Authors:** Syed Wasif Gillani, Syed Azhar Syed Sulaiman, Mohi Iqbal Mohammad Abdul, Mirza R. Baig

**Affiliations:** 1 College of Pharmacy, Taibah University, Medina, Al-Madinah Munawarah Saudi Arabia; 2 Pharmacotherapy Research Group, Islamabad, Pakistan; 3 Pharmacotherapy Research Group, Kuala Lumpur, Malaysia; 4 School of Pharmaceutical Sciences, Universiti Sains Malaysia (USM), George Town, Malaysia; 5 College of Pharmacy, University of Philippines, Quezon, Philippines; 6 Dubai College of Pharmacy, Dubai, UAE

**Keywords:** Metformin, Diabetes care, Antioxidants, Anti-inflammatory, Randomized control trials

## Abstract

**Background:**

We aimed to investigate the efficacy of ascorbic acid and acetylsalicylic acid among type II diabetes mellitus patients using metformin (only) for diabetes management therapy.

**Method:**

A 12-month single blinded multicenter randomized control trial was designed to investigate the measured variables [Glycated Hemoglobin (HbA1c), Renal function, Albumin Creatinine Ratio (ACR) etc.]. The trial was randomized into 2 experimental parallel arms (ascorbic acid vs acetylsalicylic acid) were blinded with study supplements in combination with metformin and findings were compared to control arm with metformin alone and blinded with placebo. Withdrawal criteria was defined to maintain the equity and balance in the participants in the whole trial.

**Finding:**

Patients with metformin and ascorbic acid (parallel arm I) was twice more likely to reduce HbA1c than metformin alone (control arm) in a year (OR 2.31 (95% CI 1.87–4.42) *p* < 0.001). Also Parallel arm I was ten times more likely to reduced risk factors contributing to long-term diabetes complications than participants of arm II in a year (OR 10.38 (95% CI 6.91–15.77) *p* < 0.001). In contrast, parallel arm II patients were seven times more effective to reduce the risk of expected CVD development in 10 years than arm I (OR 7.54 (95% CI 3.76–10.32) *p* < 0.001).

**Conclusions:**

The trial concluded that ascorbic acid with metformin is more effective against reducing risks for diabetes related long-term complications (including ACR).

*TRIAL details* Registration No: NTR-6100, Registry Name: Netherlands Trial Registry, URL: http://www.trialregister.nl/trialreg/admin/rctview.asp?TC=6100, Date of Registration: 20th October, 2016, Date of first Enrollment: 1 November, 2015.

**Electronic supplementary material:**

The online version of this article (doi:10.1186/s12933-017-0584-9) contains supplementary material, which is available to authorized users.

## Introduction

Diabetes mellitus (DM) is one of the most important worldwide health problems with an increasing prevalence. A figure of about 592 million is expected to be raised by 2035. To improve quality of diabetic care, clinical guidelines should be implemented [1]. Considerable resources are committed to addressing important clinical interventions connected with the amelioration of diabetic-induced micro- and macrovascular complications [2]. Under diabetic conditions, ROS are produced mainly through the glycation reaction. Oxidative stress can in turn promote glycation of hemoglobin [3] and impair the ability of β-cells of the pancreas for insulin secretion [4]. Several reports have shown that patients with low concentration of antioxidants are at increased risk of diabetes complications [5, 6]. The beneficial effect of ascorbic acid consumption in diabetes is controversial. Systematic review of observational studies and meta-analysis of RCTs identified a significant correlation between ascorbic acid as an essential micronutrient and improvements in FBS level in diabetics [7]. The idea that due to impairment of insulin secretion and ascorbate cycle in DM, ascorbic acid is necessary to optimize the insulin secretory function of the islet cells [8]. It has been speculated that decreased circulating ascorbic acid level may contribute to hyperlipidemia in diabetic patients [9]. Vitamin C deficiency, might lead to impaired transport of long-chain fatty acids into the mitochondria and subsequently more triglyceride synthesis in these patients [10]. Observational studies and meta-analysis of RCTs identified a significant correlation between ascorbic acid and improvements in FBS level in diabetics. However, yet large-scale randomized trials are needed to investigate the effect of ascorbic acid supplementation on FBS and HbA1c. It might be appropriate to suggest that diabetic subjects without contraindication of ascorbic acid intake might benefit more from taking ascorbic acid from either natural sources and/or fortified foods, and ascorbic acid supplementation [7]. Observational studies and meta-analysis of RCTs identified a significant correlation between ascorbic acid and improvements in FBS level in diabetics. However, yet large-scale randomized trials are needed to investigate the effect of ascorbic acid supplementation on FBS and HbA1c [7].

Aspirin, one of the most widely used medications worldwide. It has been used as an analgesic, antipyretic, and anti-inflammatory for a long time ago. Recently, Aspirin was found not only to be used as an anti-platelet aggregation and as a chemoprevention for cardiovascular diseases, but also it plays an important role in the reversal of obesity- and diet-induced insulin resistance [11]. As an old drug for new use, offers unique approaches for the treatment of type 2 diabetes because of its insulin-sensitizing and anti-inflammatory properties [12]. Recent studies also indicated that aspirin might be an insulin sensitizing agent and they may be used to reverse hyperglycemia, hyperinsulinemia, and dyslipidemia by sensitizing insulin signaling and improving insulin resistance [13].

Therefore, the objective of this trial was to investigate the efficacy of ascorbic acid and acetylsalicylic acid in type II diabetes mellitus patients using metformin (only) for diabetes management and preventing cardiovascular complications.

## Methods

### Trial registration

Nederland trial register (NTR)—TC6100 (20^th^ October, 2016) (WHO international clinical trial registry platform). http://www.trialregister.nl/trialreg/admin/rctview.asp?TC=6100.

### Study design

This trial is a randomized (systemic), single blinded, Multi-center, controlled, and parallel (2 arms) clinical trial. The trial was performed in compliance with the *WMA* [World Medical Association] *Declaration of Helsinki: Ethical principles for medical research involving human subjects* amended by 59th WMA (Number PHRC/HC/11/13), 2013 Seoul, Korea. Trial was approved by Clinical Research Committee (CRC) 2015, Ministry of Health (MOH), Malaysia. The trial protocol followed the Good Clinical Practice (GCP) guidelines, MOH, Malaysia.

### Study population

The study participants consist of patient diagnosed with type 2 diabetes mellitus (T2DM) and attending the outpatient department (OPD) for diabetic treatment. Participants’ eligibility criteria based on: newly diagnosed T2DM (≤5 years) with metformin (metfm) only, age ≥18 years without any other systemic and inflammatory disease (e.g., hypertension, arthritis, thyroid disorders, obesity renal impairment, pregnancy, breast feeding, cancer etc.), visiting primary health care centers for follow-up at five different locations in Pulau Pinang, Malaysia. Participants were eligible if they have glucose intolerance from last three-assessments (FBS > 7 mmol/l & Hb1Ac > 7%) and this was proven by patients’ medical history records obtained from the recruitment sites. Participants were excluded if they were using any other prescription drug or regularly using nonsteriodal anti-inflammatory drugs and documented intolerance to vitamin c and/or aspirin.

### Trial duration

The clinical trial was a 12-month (1 year) long with five-point (baseline—3 months–6 months–9 months–12 months) assessment (October 2015–August 2016).

### Intervention strategy

The study estimated the sample size of 135 per group to detect a reduction in metabolic and cardiovascular markers with p value 0.05 & a power of 80%, and also expected dropout rate of 20%. Thus, investigators aimed to recruit 450 patients. A total of 512 participants were assessed for eligibility, only 456 patients were enrolled for trial. Recruited patients were randomly (systemic) assigned to three groups, 152 patients per arm as follow (allocation ratio 1:1:1):

- Control arm (metformin + Placebo) (n = 152).
  Patients of control arm received usual metformin (Glucophage) with Placebo once daily (blinded).
- Parallel arm I (metformin + Ascorbic Acid (ACA) 500 mg) (n = 152).
  Recruited patients were given Ascorbic Acid 500 mg (Sundown vitamin c-500) once daily (Blinded) in addition to usual metformin (Glucophage) dose.
- Parallel arm II (metformin + Acetylsalicylic Acid (ASA) 100 mg) (n = 152).
  Patients received a dose of Acetylsalicylic Acid 100 mg (Apirin ^®^ Cardio) once daily in addition to usual metformin (Glucophage) dose.

*Note* Addition of placebo to control group was aimed to avoid bias in treatment with behavioural effect; if any patient from control arm accidently interact with patients’ of parallel arms (I or II).

Participants of all three groups were advices to maintain usual dietary intake as well as daily activities. Initial screening was performed for physical activity (PA) questionnaire [14]. None of the participant reported a high level of PA, majority of participants with low level and remaining at moderate level. In term of social history—smoking, participants were divided into either “ever smoke (ES)” or “never smoke (NS)”.

### Enrollment procedure

**Fig. 1:**
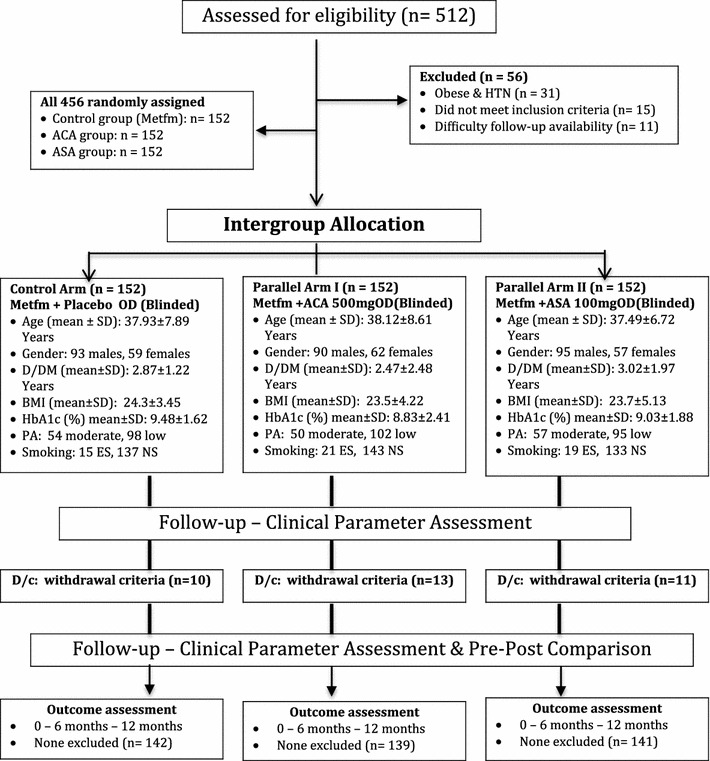
Consolidated standards of reporting trials (CONSOT) flowchart. *HTN* hypertension, *Metfm* metformin, *ACA* ascorbic acid (vitamin C), *ASA* acetylsalicylic acid (aspirin), *OD* once daily, *Blinded* single blinding = participant doesn’t know about the drug active ingredient, *D/DM* duration of diabetes mellitus, *BMI* body mass index, *PA* physical activity, *ES/NS* ever smoke/never smoke, *D/C* discontinue

Patients may be self-referred or referred through their primary physician. All eligible patients were screened to be included in this study. Eligible patients were also introduced to the study protocol by research coordinator to confirm participation (enrolled from 5 government clinics). Patients who were interested to participate in this study, required to sign a research informed consent form. Patients, who were illiterate, acquire an impartial witness to explain the study protocol before participation. Consolidated standards of reporting trials (CONSORT) flowchart can be seen in Fig. 1.

During the data collection process, all study forms were labelled with a unique study identifier. All collected forms were stored in a locked file cabinet in a locked office. Researcher will check for any missing or outlier values. All the participants monitored for 12 months, and participants needed to visit primary clinics for follow-up every month. At study baseline (Randomization), clinical assessments were performed (BMI, FBS, Hb1Ac, Waist circumference, Blood pressure, lipid profile—LDL, HDL, Triglycerides, total cholesterol), physical activity, Framingham risk score [15] and risk factors for diabetes related complications.

*Note* Treatment protocol and care services were standard all across the sites under the supervision of trial investigators (also principle investigator). All schedule appointments with the participants were actively monitored by the RCT team in collaboration with clinicians and nurses. Also all the follow-ups were monitored and validated by principle investigator, so there is no clinical/methodological bias in the procedure.

### Tools and assessments

Body Mass index (BMI): Seca Stadiometer, as Obesity is in inclusion criteria so allowed limit ≤0 kg/m^2^. Seca nonelastic tape was used to determine waist circumference (WC)

Blood Pressure (BP): Manual sphagomamometer, three readings were taken 2 min apart (mean consider at baseline)

Fasting blood sugar: An enzymatic colorimetric method with glucose oxidase was used, required normal value <5.6 mmol/l

Lipid profile: Total Cholesterol (Total-c), Triglycerides (TG), low-density lipoprotein cholesterol (LDL-c) and high-density lipoprotein cholesterol (HDL-c) were assessed by using commercial available kits

Framingham risk scale: Was used to assess the 10-year risk for development of cardiovascular disorders. Individual score was calculated as per point system and then took a mean for whole arm. Criteria: low risk ≤ 10%, moderate risk 11–19%, high-risk ≥ 20%

Risk factors for Diabetes related complications: These factors were selected from the national diabetes management guidelines; HbA1c, BP, Albumin-to-Creatinine Ratio (ACR), estimated Glomuler Filtration Rate (eGFR), lipid profile, smoking, medical follow-ups (regular), retinopathy, ethnicity. Criteria: low risk ≤1 factor, moderate risk 2 factors, high risk ≥3 factors. Individual score was calculated as per point system and then took a mean for whole arm

24/7 emergency call number: all the participants had access to 24/7-helpline number, any adverse effect/event or side effect were directly reported followed by detailed clinical examination for the possible reason.

*Note* A trained nurse of the health care centre drew a 7 ml blood sample on each visit, stored in two polyethylene evacuated tubes for quantitative measures (FBS, lipid profile, ACR and eGFR). All the qualitative measures were performed at the respective site of recruitment. Only Glycated Haemoglobin (HbA1c) evaluated every 3rd month, all the other clinical parameters assessed on monthly basis.

All the participants were assured of confidentiality clause in the research protocol. Regular reminders provided to each participant’s visit that they were participating on voluntarily basis and thus could decline at any time of study. All the positive efforts were added to minimize any potential bias and also to conduct this study in the most ethical manner possible.

### Withdrawal criteria

Following is the withdrawal criteria used to identify dropouts and manage response among participants.

- Discontinue (D/c) patient follow-ups: participant withdrew consent and/or non-cooperative.
- Participant developed condition or disease or illness that changed clinical parameters or study environment.
- Intolerance/hypersensitive to study supplements: developed condition within 4 h of study supplement administration.
- Female participants became pregnant.
- Participants were clearly instructed to not take any other OTC drugs without informing the investigator at 24/7-helpline, if investigator somehow identified the use of any medications (OTC or prescribed)/herbal supplements/multivitamin supplements in any participant lead to instant D/c from the trail.
- Study supplements were dispensed on monthly basis, so participants were required to keep a logbook and their entries were monitored. Any patient found nonadherence was removed from the trial.

*Note* All the participants received a voice reminder daily at dose time to ensure adequate adherence.

### Statistical analysis

Data analysis was made using IBM SPSS Statistics, version 22 (Armok, NY). A probability of *p* < 0.05 was considered statistically significant for all tests. Continuous variables were tested for normality; any non-normal values were categorized or transformed. All variables were analyzed using descriptive analysis. Unadjusted comparisons between study arms were made using t tests for continuous variables or Chi square tests for discrete variables. One-way ANNOA were used to assess the difference between the groups at the baseline of randomization. Independent *t*-tests were used to assess the difference between the groups during and end point of the trial. Paired *t*-tests were used to evaluate the difference within the groups.

*Note* Intention-to-treat and repeated ANNOVA are not applied in the final analysis as they are primarily used to explore the time and treatment effect however our study objectives are to “compare and estimate the clinical effects of Ascorbic acid with salicylic acid in combination with metformin among diabetes patients”, we are not aimed to compare the time and treatment effect among patients.

## Results

### Trial participants and intervention

This clinical trial was aimed to determine the effect of vitamin c (ascorbic acid) and aspirin (acetylsalicylic acid) on metabolic markers (FBS, LDL, HDL, Triglycerides etc.) of type 2 Diabetes mellitus (T2DM) in combination with metformin (diabetic medication) only. The effect was compared with control group received placebo in combination with metformin. The trial was design in way to avoid all the possible bias by blinding the study supplements (single blind with placebo, ascorbic acid & acetylsalicylic acid). A 12-month long-term follow-up was managed to evaluate the impact of combinations on risks for development of cardiovascular disorders (Framingham scale) and also on risk factors for development/progression of diabetes related complications. The progression of participants in the trail is shown in Fig. 1. Four hundred and twenty-two patients completed the study, a total of thirty-four patients were discontinue during follow-up because they met one or more of the withdrawal criteria (ten patients from control group, thirteen patients from parallel arm I and eleven patients from parallel arm II) (Fig. 1. Consolidated Standards of Reporting Trials (CONSOT) flowchart).

### Equity and balance at baseline

The baseline assessments include: demographic (age, gender, duration of diabetes history, physical activity), anthropometric (BMI, WC), clinical characteristics (HbA1c, FBS, Lipid profile, ACR, eGFR) and clinical markers (Framingham Scale, Risk factors for diabetes related complications) for quantify the long-term diabetes related complications of the participants in all three groups were compared (Table 1). Four hundred fifty-six patients were recruited at the baseline of the trial. To determine the balance in-between the groups, statistical analysis performed among all the patients’.

**Table 1:** Demographic and clinical variables at baseline (randomization) in all three arms (n = 456)

Variables	Control arm^a^ (n = 152)		Parallel arm I^b^ (n = 152)		Parallel arm II^c^ (n = 152)		*X* ^*2*^ */t*	*p* value
	Mean (SD)/percent		Mean (SD)/percent		Mean (SD)/percent			
A. Demographic								
Age (years)	37.93	(7.89)	38.12	(8.16)	37.49	(6.72)	0.08	0.87^≠^
Gender							0.02	0.13^‡^
Male	93	61.2%	90	59.2%	95	62.5%		
Female	59	38.8%	62	40.8%	57	37.5%		
B. Clinical variables								
Duration of diabetes (yrs)	2.87	(1.22)	2.47	(2.48)	3.02	(1.97)	0.07	0.36^≠^
FBS (<5.6 mmol/l)	8.13	(3.17)	7.65	(2.52)	7.71	(2.84)	0.94	0.27^≠^
HbA1c (≤5%)	9.48	(1.62)	8.83	(2.41)	9.03	(1.88)	0.71	0.45^≠^
BMI (kg/m2)	24.3	(3.45)	23.5	(4.22)	23.7	(5.13)	0.59	0.74^≠^
Male	27.1	(4.05)	28.5	(5.10)	26.6	(5.70)	1.02	0.24^≠^
Female	23.4	(6.03)	26.2	(5.96)	24.8	(5.79)	2.07	0.38^≠^
Smoker							0.26	0.16^‡^
Ever smoke (ES)	15	9.9%	21	13.9%	19	12.5%		
Never smoke (NS)	137	90.1%	143	94.1%	133	87.5%		
BP							0.11	0.29^‡^
(≤130/80 mm Hg)	12	7.9%	17	11.2%	13	8.6%		
(>130/80 mm Hg)	140	92.1%	135	88.8%	139	91.4%		
Waist circumferences (cm)	94	(10.01)	93	(10.44)	95	(12.44)	0.65	0.71^≠^
LDL-c (< 2.6 mmol/l)	3.12	(0.98)	3.09	(1.12)	3.19	(1.19)	0.21	0.48^≠^
HDL-c (1.0–1.5 mmol/l)	0.98	(1.27)	1.01	(0.87)	0.95	(1.06)	0.31	0.62^≠^
Total- c (<5.2 mmol/l)	6.11	(2.48)	5.97	(2.69)	6.07	(2.71)	0.73	0.77^≠^
Triglycerides (<1.7 mmol/l)	1.84	(1.02)	1.91	(1.12)	1.93	(1.09)	0.67	0.35^≠^
ACR (mg/mmol)*	3.79	(2.66)	3.81	(2.31)	3.80	(2.59)	0.98	0.98^≠^
eGFR (>60 ml/min/1.73 m^3^)	101.21	(15.37)	107.44	(13.84)	106.32	(15.91)	0.61	0.81^≠^
Assessment of risk for diabetes related complications^d^ (>3 factors)	Mean (SD)		Mean (SD)		Mean (SD)		0.85	0.61^≠^
	3.82 (1.81)		3.90 (1.67)		4.03 (1.79)			
	High risk		High risk		High risk			
Framingham risk score^e^	Mean (SD)		Mean (SD)		Mean (SD)		0.71	0.32^≠^
Low risk (≤10)	20.34 (3.12)		21.98 (4.41)		20.18 (4.02)			
Moderate risk (10–19)	High risk		High risk		High risk			
High risk (≥20)								
Physical activity							0.91	0.49^‡^
High	–		–		–			
Moderate	54 (35.5%)		50 (32.9%)		57 (37.5%)			
Low	98 (64.5%)		102 (67.1%)		95 (62.5%)			

*BP* blood pressure, *BMI* body mass index, *HbA1c* glycated hemoglobin, *FBS* fasting blood sugar, *LDL-c* low density lipoproteins cholesterol, *HDL-c* high density lipoprotein cholesterol, *Total-c* total cholesterol, *ACR* albumin-to-creatinine ratio, *eGFR* estimated glomular filtration rate, *IQR* interquartile range

^≠^One way—ANOVA

^‡^Chi square

^a^Metfm with placebo OD (Blinded)

^b^Metfm with ascorbic acid 500 mg OD (Blinded)

^c^Metfm with acetylsalicylic acid 100 mg OD (blinded), * Males: <2.5 mg/mmol & females: 3.5 mg/mmol

^d^Risk factors: HbA1c, BP, ACR, eGFR, Lipid profile, smoking, medication reviews (regular checkups)

^e^Framingham Risk score: Cannadian cholerterol guidelines to predict 10-years risk of developing cardiovascular disease—individualize score was calculated first then check for mean ± SD

Findings showed no significant difference among compared variables; no difference in terms of age, gender, smoking habit, physical activity or duration of diabetes history. Also, equality in-between the groups were ensured with non-significant difference of BMI & WC comparison for male and female. Similarly, no significant difference was detected when comparing the groups in term of clinical characteristics and clinical markers. Thus, such results indicate balance and equity at baseline between the groups, because the statistical significance didn’t reach the level of *p* ≤ 0.05.

### Glucose tolerance (FBS & HbA1c) pattern in the trial

Within group assessment showed significant difference with FBS from baseline to 12 months of the trial: Parallel arm I (Mean ± SD: 7.65 ± 2.52 to 5.18 ± 1.66, *p* < 0.01) and Parallel arm II (Mean ± SD: 7.71 ± 2.84 to 6.27 ± 2.19, *p* < 0.05) however no significant difference was detected in control arm (Table 2).

**Table 2:** Change in clinical variables after intervention among three arms (n = 422)

Variables	Control Arm^a^ (n = 142) Mean ± SD		Parallel arm I^b^ (n = 139) Mean ± SD		Parallel arm II^c^ (n = 141) Mean ± SD		Control versus arm I	Control versus arm II
	Baseline	12 month	Baseline	12 month	Baseline	12 month	*p* value^≠^	*p* value^≠^
FBS (<5.6 mmol/l)	8.13 ± 3.17	7.94 ± 4.10	7.65 ± 2.52	5.18 ± 1.66**	7.71 ± 2.84	6.27 ± 2.19*	0.001	0.01
HbA1c (≤5%)	9.48 ± 1.62	9.44 ± 1.99	8.83 ± 2.41	6.45 ± 1.21**	9.03 ± 1.88	6.93 ± 1.58**	0.001	0.01
BMI (kg/m2)	24.3 ± 3.45	25.1 ± 4.01*	23.5 ± 4.22	24.1 ± 3.45	23.7 ± 5.13	24.4 ± 5.18	0.61	0.78
Male	27.1 ± 4.05	28.5 ± 3.87	28.5 ± 5.10	28.9 ± 4.08	26.6 ± 5.70	27.1 ± 4.34	0.45	0.53
Female	23.4 ± 6.03	25.9 ± 5.47*	26.2 ± 5.96	25.67 ± 4.29	24.8 ± 5.79	25.4 ± 5.12	0.39	0.42
BP							0.01^‡^	0.01^‡^
(≤130/80 mm Hg)	12–7.9%	25–16.5%	17–11.2%	88–57.9%	13–8.6%	102–67.1%		
(>130/80 mm Hg)	140–92.1%	127–83.5%	135–88.8%	64–42.1%	139–91.4%	50–32.9%		
LDL-c (<2.6 mmol/l)	3.12 ± 0.98	3.56 ± 1.17*	3.09 ± 1.12	2.47 ± 2.11*	3.19 ± 1.19	2.16 ± 0.78**	0.03	0.001
HDL-c (1.0–1.5 mmol/l)	0.98 ± 1.27	0.76 ± 1.02*	1.01 ± 0.87	1.37 ± 1.03*	0.95 ± 1.06	1.39 ± 1.11*	0.01	0.001
Total- c (<5.2 mmol/l)	6.11 ± 2.48	6.44 ± 3.65**	5.97 ± 2.69	5.07 ± 1.49*	6.07 ± 2.71	4.8 ± 1.89**	0.04	0.001
Triglycerides (<1.7 mmol/l)	1.84 ± 1.02	2.11 ± 1.78*	1.91 ± 1.12	1.28 ± 0.67*	1.93 ± 1.09	1.16 ± 0.73**	0.02	0.001
ACR (mg/mmol)	3.79 ± 2.66	3.81 ± 2.03	3.81 ± 2.31	2.32 ± 1.98**	3.80 ± 2.59	3.22 ± 2.06	0.001	0.45
eGFR (>60 ml/min/1.73 m^3^)	101.21 ± 15.37	97.89 ± 18.20*	107.44 ± 13.84	106.71 ± 13.01	106.32 ± 15.91	107.86 ± 14.37	0.21	0.37
Assessment of risk for diabetes related complications^d^ (>3 factors)	High risk Mean (SD)	High risk Mean (SD)	High risk Mean (SD)	Low risk Mean (SD)	High risk Mean (SD)	Mod risk Mean (SD)	0.001	0.01
	3.82 (1.81)	4.01* (2.03)	3.90 (1.67)	0.82** (1.01)	4.03 (1.79)	1.67** (0.88)		
Framingham risk score^e^								0.001
Low risk (≤10)	High risk	High risk	High risk	High risk	High risk	Low risk		
Moderate risk (10–19)	20.34 ± 3.12	21.42 ± 2.65	21.98 ± 4.41	11.69 ± 2.99**	20.18 ± 4.02	7.23 ± 1.22**	0.01	
High risk (≥20)								
Physical activity							0.04^‡^	0.01^‡^
High	–	–	–	15–9.9%	–	19–12.5%		
Moderate	54–35.5%	69–45.4%	50–32.9%	71–46.7%	57–37.5%	69–45.4%		
Low	98–64.5%	83–54.6%	102–67.1%	66–43.4%	95–62.5%	64–42.1%		

*BP* blood pressure, *BMI* body mass index, *HbA1c* glycated hemoglobin, *FBS* fasting blood sugar, *LDL-c* low density lipoproteins cholesterol, *HDL-c* high density lipoprotein cholesterol, *Total-c* total cholesterol, *ACR* albumin-to-creatinine ratio: Males: <2.5 mg/mmol and females: 3.5 mg/mmol, *eGFR* estimated glomular filtration rate, *IQR* interquartile range, *Mod* moderate

* *p* < 0.05

** *p* < 0.01 (two-tailed) (paired-samples *t* test)

^≠^Independent-samples *t* test

^‡^Chi square

^a^Metfm with Placebo OD (Blinded)

^b^Metfm with Ascorbic Acid 500 mg OD (Blinded)

^c^Metfm with Acetylsalicylic Acid 100 mg OD (Blinded)

^d^Risk factors: HbA1c, BP, ACR, eGFR, Lipid profile, smoking, medication reviews (regular checkups)

^e^Framingham Risk score: Cannadian cholerterol guidelines to predict 10-years risk of developing cardiovascular disease—individualize score was calculated first then check for mean ± SD

**Fig. 2:**
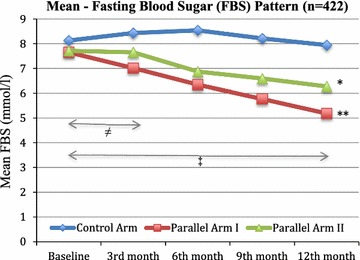
Fasting blood sugar (FBS) distribution pattern in three groups during trial. Control Arm (metformin + Placebo) n = 142, Parallel Arm I: metformin + Ascorbic acid 500 mg OD: n = 139, Parallel Arm II: metformin + Acetylsalicylic acid 100 mg OD: n = 141, FBS: Fasting blood sugar, * *p* < 0.05, **p < 0.01 independent-samples *t* test: comparison between interventional arms (I & II) versus Control arm. ^≠^ *p* < 0.05, ^‡^ *p* < 0.01 independent-samples *t* test: comparison Parallel arm I versus Parallel arm II

Intergroup assessment showed significant difference between control arm and parallel arm I (*p* < 0.001), similar pattern was observed with control arm versus parallel arm II (*p* < 0.01). Comparison between parallel arm I with parallel arm II from baseline to 3 months showed mild significance (*p* < 0.05) however as trial progressed highly significant (*p* < 0.01) difference was observed at 12th month (end of trial) (Fig. 2). Data was transform to estimate the difference between parallel arm I and II, Odd ratios (OR) showed that patients with ascorbic acid (parallel arm I) were three times more likely to reduce FBS than patients with acetylsalicylic acid (parallel arm II) (OR 3.44 (95% CI 1.60–5.68) *p* < 0.001).

Similar pattern was observed with HbA1c, significant reduction was observed within the group with both parallel arm I and II (*p* < 0.001 and *p* < 0.001), Mean reduction of HbA1c was 2.78 (parallel arm I) versus 2.24 (parallel arm II). Findings also suggested that patients with metformin and ascorbic acid (parallel arm I) were two times more likely to reduce HbA1c than metformin alone (control arm) in a year (OR 2.31 (95% CI 1.87–4.42) *p* < 0.001).

### Effect of trial on anthropometric values

Neither ascorbic acid nor acetylsalicylic acid showed any significant change (reduction or increase) to anthropometric values both within the group and intergroup (compare to control) (Table 2).

### Effect of trial supplements on Lipid profile

Within group assessment showed that mean reduction of LDL-c was significantly (*p* < 0.001) reduced to (2.16) from (3.19) among patients receiving acetylsalicylic acid (parallel arm II), mild significance (*p* < 0.05) was also associated with parallel arm I. In contrast participants receiving metformin (control arm) alone showed significant (*p* < 0.05) increase in value to (3.56) from (3.12).

Intergroup assessment presented significant difference between parallel arm I & II compared to control arm (*p* < 0.03 and *p* < 0.001). Similar patterns were observed with other lipid parameters (Table 2).

Participants of parallel arm II were two times more likely to reduce LDL-c as compared to participants of parallel arm I in a year (OR 2.06 (95% CI 1.21–4.78) *p* < 0.001).

HDL-c values were increased at the same level among the participants of both parallel arms (OR 1.04 (95% CI 0.16–1.77) *p* < 0.91). Similar pattern was observed with TG (triglycerides) in comparison between parallel arm I and parallel arm II. However patients with acetylsalicylic acid (parallel arm II) were four times more likely to reduce Total- c (total cholesterol) as compared to patients received metformin alone (control arm) in a year (OR 4.35 (95% CI 2.19–7.46) *p* < 0.001).

### Changes in ACR & eGFR estimates

Albumin-to-creatinine ratio (ACR) value was significantly (*p* < 0.001) reduced within ascorbic arm during the trial, both control arm and acetylsalicylic arm didn’t show any difference as compared to baseline. It was estimated that participants with ascorbic supplement in combination with metformin (parallel arm I) is five times more likely to reduce the mean ACR value than metformin alone (control arm) in a year (OR 5.16 (95% CI 2.81–7.93) *p* < 0.001).

eGFR; Participants of ascorbic acid and acetylsalicylic acid were exhibited no significant difference from baseline to endpoint within a group assessment. Also intergroup assessment showed no difference among all three arms. However, patients with metformin alone (control arm) showed mild significant reduction in eGFR (*p* < 0.05) from baseline to endpoint of trial suggesting possible deterioration of renal function due to glucose intolerance overtime (Table 2).

### Risk factors for developing Diabetes related long- term complications

**Fig. 3:**
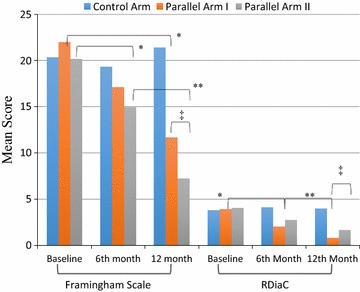
Pattern of Framingham mean score and risks for diabetes related complications in the trial with ascorbic acid arm and acetylsalicylic acid arm versus control arm (n = 422). Control Arm (metformin + Placebo) n = 142, Parallel Arm I: metformin + Ascorbic acid 500 mg OD: n = 139, Parallel Arm II: metformin + acetylsalicylic acid 100 mg OD: n = 141, RDiaC: Risk for diabetes related complications, *p < 0.05, **p < 0.01 independent-samples *t* test: comparison between interventional arms (I & II) versus Control arm, ^‡^ *p* < 0.01 independent-samples *t* test: comparison Parallel arm I versus Parallel arm II

The interventional arms (I & II) were showed significant (*p* < 0.001) reduction of risk factors contributing to long-term diabetes related complications from baseline to endpoint (Table 2). Intergroup assessment between parallel arm I and arm II suggested that participants with arm I were ten times more likely to reduced risk factors contributing to long-term diabetes complications than participants of arm II in a year (OR 10.38 (95% CI 6.91–15.77) *p* < 0.001) (Fig. 3).

### Change of Framingham risk score respective to trial supplement

The study supplements showed significant association with the reduction of Framingham score in the trial compared with baseline; arm I (*p* < 0.001, moderate): arm II (*p* < 0.001, Low), however control arm presented an increase in mean score sustaining ‘high risk’ (Table 2). Comparison between arm I & arm II to estimate the impact of effect showed that patients with acetylsalicylic arm II were seven times more effective to reduce the risk of expected CVD development in 10 years than ascorbic arm I (OR 7.54 (95% CI 3.76–10.32) *p* < 0.001) (Fig. 3).

### Adverse drug reactions/events reported via 24/7-helpline

A total of 133 diabetes related complications/complaints were reported in a year, among them control arm (89, 66.9%), parallel arm I (23, 17.3%) and rest 21 (15.8%) from parallel arm II. Followings were the distribution pattern with the study arms; Missed dose: 25 (18.8%) with Control arm only.

Hypoglycemic episodes: 30 (22.6%) with Control arm, 2 (1.5%) with Parallel arm I, and 3 (2.3%) with Parallel arm II).

Hyperglycemic episodes: 45 (33.8%) Control arm, 3 (2.3%) Parallel arm I, and 5 (3.7%) with Parallel arm II.

Wrong timing of medication intake: 11 (8.2%) Control arm, 5 (3.7%) Parallel arm I, and 4 (3.0% Parallel arm II).

Above finding clearly suggested increased number of diabetes related medication nonadherence and intolerance complaints with control arm as compared with parallel arm I and II. Results reflected the reduced nonadherence and intolerance episodes with both ascorbic and acetylsalicylic arm in a year.

## Discussion

Generally scientific literature suggested decrease anti-oxidative system of the body with diabetes mellitus over period of time, thus increased the oxidative load [16]. Several observational epidemiological studies reported the increase risks of diabetes related complications with low level of antioxidants [6, 17]. Some experimental researches also supported the oxidative stress role in pathogenesis of diabetes and related complications [18, 19].

This influenced the scientific community from all across the globe to determine the correlational pattern between diabetes management and antioxidants. So, numerous human and animal studies were performed over last two decades, to estimate the positive effects of antioxidant therapy on oxidative stress status [via Reactive Oxygen species (ROS)], relative influence on *b*-cell mass and insulin secretion [19–21]. However, antioxidants beneficial role on insulin resistance/sensitivity pattern is far from exclusive [22, 23]. Also Oral Ascorbic acid supplementation ameliorates skeletal muscle oxidative stress during hyperinsulinemia and improves insulin-mediated glucose disposal in people with type 2 diabetes [24].

Hydrophilicity and concentration dependent action of Vitamin C (ascorbic acid) could have both pro- and antioxidative functions [25]. It is believed that ascorbic acid optimizes the islets’ insulin secretion, to facilitate impaired insulin secretion and ascorbate cycle in diabetes mellitus [8].

There are several in vitro and in vivo studies to determine the influence of antioxidants in diabetes management. Chen and colleges [26] conducted a RCT with T2DM patients including 800 mg ascorbic acid interventional arm and compared to Placebo over a period of 4 weeks, findings showed nonsignificant effects to glucose tolerance parameters (FBS etc.). In contrast, Evans and colleagues [27] conducted similar RCT with T2DM patients but increase the ascorbic acid dose to 1000 mg and compared to Placebo over a period of 6 weeks, results showed significant decrease in TG, increase in HDL-c, decrease in SBP&DBP and also FBS levels with ascorbic acid group compared with placebo. Likewise, Paolisso and team [28] also conducted a RCT in T2DM patients including ascorbic acid 1000 mg versus Placebo over 16 weeks of follow-up, findings suggested significant decrease in TG, LDL-c, HbA1c, FBS also whole body glucose disposal with ascorbic acid group compared with placebo. These trials limited with the standardization of hyperglycemic agents used by trial participants and also the short duration and high dose of ascorbic acid. The high doses of ascorbic acid have adverse effects such as oxidative damage to DNA and decrease NO bioactivity [29]. As discussed early ascorbic acid exhibits concentration-dependent action to antioxidant functions, and significant reduction in diabetes assessment parameters (FBS, HbA1c etc.) are not truly the effects of ascorbic acid alone; other hyperglycemic agents in combination to antioxidants also influence the value of assessment parameters [30, 31]. Thus control the use of hyperglycemic medications in the trial participants would provide a true picture of ascorbic acid effect in diabetes.

This trial showed significant reduction in FBS, HbA1c, Diabetes related long-term complications, LDL-c, TG, Total-c, also Framingham score for 10-years risk of developing CVD with ascorbic acid group compared to control group. This trial benefitted with the 48 weeks (long term follow up) as compared with 4–12 weeks trails before; also controlled the impact of hyperglycemic agents by standardizing metformin use only by all the trial participants (interventional arms and control arm). All the previous literature [17, 26] lacked to provide the combination effect of metformin with ascorbic acid. Similarly, other trials only discussed the effect of ascorbic acid on the management of current diabetes assessment parameters, none correlates the effect/impact of ascorbic acid use on long-term diabetes related complications & development of CVDs over time. Also among type 2 diabetic patients, low levels of serum vitamin C were closely associated with concomitant renal dysfunction and low-grade inflammation [32].

However recent meta-analysis didn’t support the beneficial effect of antioxidants on homoeostatic model assessment index (HOMA) and also identified the lack of evidence on long-term use of antioxidants in diabetes [33, 34]. Thus, this trial answered the limitations of meta-analysis report by comparing low dose antioxidants (500 mg) with metformin for long duration of time (48 weeks) against metformin with placebo. This trial also reported that antioxidants combination with metformin (only) improved the glucose tolerance and reduced disease progression, these findings consistent with recent in vitro studies [33–35] and ADA recommendations [36].

Recent interest on the theory of chronic low-grade inflammation contributed to pathogenesis of insulin resistance and T2DM provided credible hypothesis to establish the link, thus targeting the inflammatory pathway might enhance the pharmacological response for the treatment of T2DM [30, 37–39]. Safety profile of salicylates, provided wide room for clinical studies to investigate the response in patient with T2DM and also obese non-diabetic individuals [40–45]. Cardiovascular events are the chronic complications of T2DM and aspirin is widely used to reduce such events. Therefore, this trial further investigates the efficacy of aspirin on T2DM and its complications. Furthermore, since aspirins are inexpensive, using salicylates for treatment of T2DM would have potential health-economic benefit worldwide [33, 34]. Derosa et al, also reported that the use of ASA in primary prevention could be useful in patients with type 2 diabetes mellitus and hypertension [46].

Several trials in the last decade were conducted to determine the effect of aspirin in diabetes pathogenesis [47–53]. A recent meta-analysis conducted by Fang and colleagues [54] concluded that the anti-diabetic effect of salicylates is in a dose-dependent manner. High doses of salicylates may have beneficial effects on reducing FPG, HbA1c level and increasing fasting insulin concentration, and may also have some positive effects on lipidemia and inflammation-associated parameters for patients with T2DM, without severe adverse effects. It is also reported in the literature that Biomarkers of inflammation and endothelial dysfunction were positively associated with incident T2D [55, 56]. All the previous trials were limited to:

- None of the trials have determined the combine drug effects (metformin with aspirin).
- Comorbidities were not controlled among all the trial participants (diverse data).
- Neither of the trials has compared the inflammatory effect of aspirin with antioxidant effect of ascorbic acid (control variable: diabetic medication, metformin) nor determines the better treatment option.
- Also, effect of aspirin on long-term diabetes related complications, lipid profile, and Framingham scale for risks of CVD development never evaluated before.

This trial manage to standardize the use of metformin (alone) for diabetes in all the participants, also all the patients with any systemic or informatory diseases (including pregnancy) were excluded in the early recruitment phase to avoid impact of disease on relative assessment variables. Previous literature suggested that high dose >1000 mg/day significantly reduced the FBS [47], but this trial reported significant reduction of FBS within 3 months with aspirin 100 mg/day (parallel arm II) compared with control (metformin only). Thus preventing adverse drug reactions with high doses, e.g., gastrointestinal discomforts, extremity edema, hemorrhage risks etc. Low doses of aspirin are widely used to prevent cardiovascular events in high-risk patients [55, 56]. The results of our trail showed that low doses of aspirin had significantly positive effects on glucose metabolism, lipidemia and reduced CVD development risks in patients with T2DM. Aspirin improves the glucose metabolism for patients with T2DM through inhibiting IKKβ/NF-κB axis. However, so far no trial have used high doses of aspirin to reduce cardiovascular events, this is the first trial that determine and estimate the reduction of cardiovascular event by using Framingham scale.

Most importantly scientific literature is diverse with the antioxidants versus anti-inflammatory agents use in diabetes; several trials have shown beneficial outcomes with both. But none of them have compared the ascorbic acid versus aspirin in RCT to determine benefits with glucose metabolism, lipid profile and reduction in both CVD risks and risks for long-term diabetes related complications (Additional file 1). Type 2 diabetic patients who were started on metformin monotherapy showed improvement in many of the clinical parameters and a reduction in all-cause mortality and CVD events than lifestyle modifications alone [57, 58]. If there is no contraindication and if tolerated, diabetic patients should be prescribed with metformin early in the course of the diabetic management to minimize their risk of having the cardiovascular events and mortality in the long run [58–62]. Lastly, differences of HDL-C levels in response to metformin by race/ethnicity, and that racial and ethnic identity is a factor to consider when interpreting the effects of metformin treatment [63].

## Conclusions

The strength of our study lies in the comparison between interventional arms and control group at the end point of the study without violating any patients’ characteristics, so equality was assumed in all aspects. To the best of our knowledge, no previous studies have evaluated comparison of clinical efficacy of ascorbic acid and aspirin in combination with metformin. Our findings indicate significant improvement in glucose metabolism and lipid-profile variables with both interventional arms (ascorbic acid vs aspirin). The trial concluded that ascorbic acid with metformin is more effective against reducing risks for diabetes related long-term complications (including ACR), however if the treatment goal is to reduce cardiovascular events or risks for CVD development then aspirin with metformin is benefitted over ascorbic acid.

Further RCT studies with different diabetic medications are needed to explore the efficacy of antioxidants in diabetes with comorbidities.

## Additional file

**Additional file 1.** Improving clinical care of diabetes mellitus; randomized clinical trial.
