# Supplementary material for: Combined effect of metformin with ascorbic acid versus acetyl salicylic acid on diabetes-related cardiovascular complication; a 12-month single blind multicenter randomized control trial
Source: Cardiovasc Diabetol. 2017 Aug 14;16:103. doi: 10.1186/s12933-017-0584-9 (PMC5556597; doi:10.1186/s12933-017-0584-9)
Supplement: Supplementary file 1 — Additional file 1. Improving clinical care of diabetes mellitus; randomized clinical trial. [file 12933_2017_584_MOESM1_ESM.pdf]

Protocol Detailing

TITLE:

Improving clinical care of diabetes mellitus; randomized clinical trial

PROTOCOL Identifying Number: Met_AA:15-01

PRINCIPLE Investigator: Dr. Syed Wasif Gillani

IDN Sponsor: Dr. Syed Wasif Gillani, Prof. Syed Azhar Syed Sulaiman

Funded by: Dr. Syed Wasif Gillani

Version no: v.1.0

01 October 2015

Schematic of Study Design

1 June – 30 August: Recruitment

Total: 512
Obtain Informed consent form
Screen potential Subjects by inclusion and exclusion criteria
Randomization
Interventional groups (2 arms): 152 per arm
Control group = 152

1 October 2015: Screening & Baseline

Clinical Screening: Baseline assessments

Obtain history, documents

Initiate study intervention

1 Jan 2015: Follow-up Assessment of study endpoints

Clinical Screening: Baseline assessments

Withdrawal criteria

1 April 2015: Follow-up Assessment of study endpoints

Clinical Screening: Baseline assessments

Withdrawal criteria

1 July 2015: Follow-up Assessment of study endpoints

Clinical Screening: Baseline assessments

Withdrawal criteria

30 September 2015: Follow-up Assessment of study endpoints

Completed n = 422,

Withdrawal n = 34

Clinical Screening: Baseline assessments

Withdrawal criteria

STUDY DESIGN AND ENDPOINTS

Description

This is a randomized (systemic), single blinded, Multi-center, controlled, and parallel (2 arms) clinical trial.

a. Study supplements: Ascorbic acid Vs Acetylsalicylic acid in combination with metformin. Study agents were blinded in the interventional arms however placebo (blinded) used with control arm.
b. No dose escalations (standard dosing)
c. Monitoring of ADE, ADR, SE by registering calls on 27/4 helpline and each follow-up assessment.

Endpoints

Primary Outcomes:

a. Improved glucose metabolism
b. Reduction in lipid metabolic markers

Secondary outcomes:

a. Reduction in diabetes related complications over a period of 12 months
b. Improved clinical outcomes in lipid profiling
c. Framingham scale assessment for risk of coronary artery disease risks
d. Combine effect of metformin with antioxidants and anti-inflamatory

Study Enrollment and recruitment

Principle investigator was sole responsible for recruitment, randomization and assignment to intervention. Patients may be self-referred or referred through their primary physician. All eligible patients were screened to be included in this study. Eligible patients were also introduced to the study protocol by research

coordinator to confirm participation (enrolled from 5 government clinics). ***Patients who were interested to participate in this study, required to sign a research informed consent form. Patients, who were illiterate, acquire an impartial witness to explain the study protocol before participation. Consolidated standards of reporting trials (CONSORT) flowchart can be seen in Figure 1.***

During the data collection process, all study forms were labelled with a unique study identifier. All collected forms were stored in a locked file cabinet in a locked office. Researcher will check for any missing or outlier values. All the participants monitored for 12 months, and participants needed to visit primary clinics for follow-up every month. At study baseline (systemic random sampling), clinical assessments were performed (BMI, FBS, Hb1Ac, Waist circumference, Blood pressure, lipid profile – LDL, HDL, Triglycerides, total cholesterol), physical activity, Framingham risk score [15] and risk factors for diabetes related complications.

Study Population

The study participants consist of patient diagnosed with type 2 diabetes mellitus (T2DM) and attending the outpatient department (OPD) for diabetic treatment. **Participants' eligibility criteria** based on: newly diagnosed T2DM (≤ 5 years) with metformin (metfm) only, age ≥ 18 years without any other systemic and inflammatory disease (e.g., hypertension, arthritis, thyroid disorders, obesity renal impairment, pregnancy, breast feeding, cancer etc), visiting primary health care centers for follow-up at five different locations in Pulau Pinang, Malaysia.

Participants were eligible if they have glucose intolerance from last three-assessments (FBS > 7 mmol/l & Hb1Ac >7 %) and this was proven by patients' medical history records obtained from the recruitment sites. **Participants were excluded** if they were using any other prescription drug or regularly using nonsteriodal anti-inflammatory drugs and documented intolerance to vitamin c and/or aspirin.

Retention strategies

Participants were provided with incentives: (cash vouchers (RM 100)) on each assessment follow-up.

Food, transportation was provided to reduce transportation barrier.

Free metformin supply during study duration.

Monthly telecommunication reloads of RM 50 for emergency access to 24/7 helpline.

Monthly visit reminders were sent to participants to increase participant retention.

Participants Withdrawal or Termination criteria

Following is the withdrawal criteria used to identify dropouts and manage response among participants.

a. Discontinue (D/c) patient follow-ups: participant withdrew consent and/or non-cooperative.

b. Participant developed condition or disease or illness that changed clinical parameters or study environment.

c. Intolerance/hypersensitive to study supplements: developed condition within 4 hours of study supplement administration.

d. Female participants became pregnant.

e. Participants were clearly instructed to not take any other OTC drugs without informing the investigator at 24/7-helpline, if investigator somehow identified the use of any medications (OTC or prescribed) / herbal supplements / multivitamin supplements in any participant lead to instant D/c from the trail.

f. Study supplements were dispensed on monthly basis, so participants were required to keep a logbook and their entries were monitored. Any patient found nonadherence was removed from the trial.

Note: All the participants received a voice reminder daily at dose time to ensure adequate adherence. Replacements are not allowed in the trial.

Suspension of the study Clause:

If the study is prematurely terminated or suspended, the PI will promptly inform the IRB and will provide the reason(s) for the termination or suspension. Circumstances that may warrant termination or suspension include, but are not limited to:

- Determination of unexpected, significant, or unacceptable risk to participants
- Demonstration of efficacy that would warrant stopping
- Insufficient compliance to protocol requirements
- Data that are not sufficiently complete and/or evaluable
- Determination of futility Study may resume once concerns about safety, protocol compliance, data quality are addressed and satisfy the sponsor, MOH, CRC.

Study Agents

Acquisition

Both ascorbic Acid and Acetylsalicylic acid were acquired by investigator from the retail pharmacies, however metformin acquired from the relevant pharmacy at the site of recruitment and follow-up meetings.

Dosing and administration

Study agents were acquired in Oral tablet form, administration was preferred with metformin, and however only once daily dosing is preferred.

Dosing and Duration

a. Control arm (metformin + Placebo) (n =152)

Patients of control arm received usual metformin (Glucophage) with Placebo once daily (blinded).

b. Parallel arm I (metformin + Ascorbic Acid (ACA) 500 mg) (n=152)

Recruited patients were given Ascorbic Acid 500mg (Sundown vitamin c-500) once daily (Blinded) in addition to usual metformin (Glucophage) dose.

c. Parallel arm II (metformin + Acetylsalicylic Acid (ASA) 100mg) (n=152)

Patients received a dose of Acetylsalicylic Acid 100mg (Apirin ® Cardio) once daily in addition to usual metformin (Glucophage) dose.

Note: Addition of placebo to control group was aimed to avoid bias in treatment with behavioural effect; if any patient from control arm accidently interact with patients' of parallel arms (I or II).

Study Specific Procedure

a. Medical history (obtained from both by interview and from medical records)
b. Clinical Assessments were performed with the help of registered nurse and certified diagnostic laboratory.
c. Biological Specimen collection and transportation is briefly explained in methods section of the manuscript.
d. Participants received regular feedback on clinical assessments.
e. Each follow-up assessment, participants had to attend 5-10min counseling session with principle investigator.
f. Assessment of Study supplement adherence done by various tools.
g. Logbook was provided to each participant for self-notes.

Tools and Assessments

Body Mass index (BMI): Seca Stadiometer, as Obesity is in inclusion criteria so allowed limit = < 30kg/m^2. Seca nonelastic tape was used to determine waist circumference (WC).

Blood Pressure (BP): manual sphagomamometer, three readings were taken 2 minutes apart (mean consider at baseline).

Fasting blood sugar: an enzymatic colorimetric method with glucose oxidase was used, required normal value < 5.6mmol/l.

Lipid profile: Total Cholesterol (Total-c), Triglycerides (TG), low-density lipoprotein cholesterol (LDL-c) and high-density lipoprotein cholesterol (HDL-c) were assessed by using commercial available kits.

Framingham risk scale: used to assess the 10-year risk for development of cardiovascular disorders. Individual score was calculated as per point system and then took a mean for whole arm. Criteria: low risk ≤ 10 %, moderate risk 11-19%, high-risk ≥ 20%.

Risk factors for Diabetes related complications: these factors were selected from the national diabetes management guidelines; HbA1c, BP, Albumin-to-Creatinine Ratio (ACR), estimated Glomuler Filtration Rate (eGFR), lipid profile, smoking, medical follow-ups (regular), retinopathy, ethnicity. Criteria: low risk ≤ 1 factor, moderate risk 2 factors, high risk ≥ 3 factors. Individual score was calculated as per point system and then took a mean for whole arm.

24/7 emergency call number: all the participants had access to 24/7-helpline number, any adverse effect/event or side effect were directly reported followed by detailed clinical examination for the possible reason.

Note: A trained nurse of the health care centre drew a 7ml blood sample on each visit, stored in two polyethylene evacuated tubes for quantitative measures (FBS, lipid profile, ACR and eGFR). All the qualitative measures were performed at the respective site of recruitment. Only Glycated Haemoglobin (HbA1c) evaluated every 3rd month, all the other clinical parameters assessed on monthly basis.

All the participants were **assured of confidentiality clause** in the research protocol.

Regular reminders provided to each participant's visit that they were participating on voluntarily basis and thus could decline at any time of study.

All the positive efforts were added to minimize any potential bias and also to conduct this study in the most ethical manner possible.

Assessment of Safety

The study is conducted at multiple sites, and will require centralized safety oversight.

Safety Parameters

Adverse Events (AEs): Adverse event means any untoward medical occurrence associated with the use of an intervention in humans, whether or not considered intervention-related.

Serious Adverse Effects (SAE): an unanticipated adverse device effect means any serious adverse effect on health or safety or any life-threatening problem or death caused by, or associated with, a device, if that effect, problem, or death was not previously identified in nature, severity, or degree of incidence in the investigational plan or application (including a supplementary plan or application), or any other unanticipated serious problem associated with a device that relates to the rights, safety, or Welfare of subjects.

Unanticipated problems (UP): An incident, experience, or outcome that meets the definition of an UP generally will warrant consideration of changes to the protocol or consent in order to protect the safety, welfare, or rights of participants or others. Other UPs may warrant corrective actions at a specific study site.

Corrective Actions:

a. Modification of inclusion or exclusion criteria to mitigate the newly identified risks

b. Implementation of additional safety monitoring procedures

c. Suspension of enrollment of new participants or halting of study procedures for enrolled participants

d. Modification of informed consent documents to include a description of newly recognized risks

e. Provision of additional information about newly recognized risks to previously enrolled participants

Classification of Adverse Event

Mild – Events require minimal or no treatment and do not interfere with the participant's daily activities.

Moderate – Events result in a low level of inconvenience or concern with the therapeutic measures. Moderate events may cause some interference with functioning.

Severe – Events interrupt a participant's usual daily activity and may require systemic drug therapy or other treatment. Severe events are usually potentially life threatening or incapacitating.

There is not a reasonable possibility that the administration of the study supplements caused the event, there is no temporal relationship between the study agent and event onset, or an alternate etiology has been established.

.

STATISTICAL ANALYSIS

a. Determination of sample size
b. General Approach
c. Analysis of Primary endpoints
d. Analysis of secondary endpoints
e. Safety analysis
f. Baseline descriptive analysis

Analytical Plans

a. Baseline equity and balance
b. Baseline primary endpoints to Final assessment primary endpoints
c. Baseline secondary endpoints to final assessment secondary endpoints
d. Correlational patterns between arms
e. Effect size to the secondary outcomes

Statistical Hypothesis

H01: Antioxidants combination will significantly improve the glucose metabolism as compared to metformin alone (with placebo) (baseline – to – final)

H02: Anti-inflammatory combination will significantly reduce the cardiovascular risk factors as compared to metformin alone (with placebo) (baseline – to – final)

Statistical methods:

A probability of $p<0.05$ was considered statistically significant for all tests. Continuous variables were tested for normality; any non-normal values were

categorized or transformed.

All variables were analyzed using descriptive analysis. Unadjusted comparisons between study arms were made using t-tests for continuous variables or chi-square tests for discrete variables.

One-way ANNOA were used to assess the difference between the groups at the baseline of randomization.

Independent *t*-tests were used to assess the difference between the groups during and end point of the trial.

Paired *t*-tests were used to evaluate the difference within the groups.

Ethics Protection

The trial was performed in compliance with the *WMA* [World Medical Association] *Declaration of Helsinki: Ethical principles for medical research involving human subjects* amended by 59th WMA (number PHRC/HC/11/13), 2013 Seoul, Korea. Trial was approved by Clinical Research Committee (CRC) 2015, Ministry of Health (MOH), Malaysia. The trial protocol followed the Good Clinical Practice (GCP) guidelines, MOH, Malaysia.

Data Collection and Management process

Data collection is the responsibility of the clinical trial staff at the site under the supervision of the site PI. The investigator is responsible for ensuring the accuracy, completeness, legibility, and timeliness of the data reported.

All source documents should be completed in a neat, legible manner to ensure accurate interpretation of data. Black ink is required to ensure clarity of reproduced

copies. When making changes or corrections, cross out the original entry with a single line, and initial and date the change.

DO NOT ERASE, OVERWRITE, OR USE CORRECTION FLUID OR TAPE ON THE ORIGINAL.

Copies of the electronic CRF (eCRF) are provided for use as source documents and maintained for recording data for each participant enrolled in the study. Data reported in the eCRF derived from source documents should be consistent with the source documents or the discrepancies should be explained and captured in a progress note and maintained in the participant's official electronic study record.

Study Record Retention

Study documents should be retained for a minimum of 3 years after the last Publication.

These documents should be retained for a longer period, however, if required by local regulations. No records will be destroyed without the written consent of the sponsor, if applicable. It is the responsibility of the sponsor to inform the investigator when these documents no longer need to be retained.

Protocol Deviation

A protocol deviation is any noncompliance with the clinical trial protocol, GCP, or MOH requirements.

The noncompliance may be either on the part of the participant, the investigator, or the study site staff. As a result of deviations, corrective actions are to be developed by the site and implemented promptly.

APPENDIX

Version	Date	Significant Revisions
